# Human Rabies during the COVID-19 Pandemic: Insights into Rabies Worldwide and Brazil

**DOI:** 10.1590/0037-8682-0520-2023

**Published:** 2024-02-12

**Authors:** Luís Arthur Brasil Gadelha Farias, Iusta Caminha, Lauro Vieira Perdigão, Luciano Pamplona de Góes Cavalcanti

**Affiliations:** 1 Universidade de São Paulo, Hospital das Clínicas e Laboratório de Investigação Médica - LIM 49, Departamento de Doenças Infecciosas, São Paulo, SP, Brasil.; 2 Universidade Federal do Ceará, Departamento de Saúde Comunitária, Fortaleza, CE, Brasil.; 3 Hospital São José de Doenças Infecciosas, Fortaleza, CE, Brasil.; 4 Centro Universitário Christus, Faculdade de Medicina, Fortaleza, CE, Brasil.; 5 Universidade Federal do Ceará, Programa de Pós-graduação em Patologia, Fortaleza, CE, Brasil.; 6 Universidade de Fortaleza, Fortaleza, CE, Brasil.

**Keywords:** Human Rabies, COVID-19 Pandemic, Epidemiology, Rabies virus, SARS-CoV-2

## Abstract

Human Rabies (HR) is a fatal zoonotic disease caused by lyssaviruses, with the rabies virus (RABV) identified as the causative agent. While the incidence of HR transmitted by dogs has decreased in Latin America, there has been a corresponding rise in transmission via wild animals. Given the lack of effective treatments and specific therapies, the management of HR relies on the availability of post-exposure prophylaxis and animal control measures. This review examines the dynamics and spread of HR during the global pandemic.

## INTRODUCTION

Human rabies (HR) is a highly lethal zoonotic viral disease that is preventable with vaccines and immunoglobulin^1^. HR manifests as acute progressive encephalitis caused by the rabies virus (RABV), a single-stranded RNA virus belonging to the Rhabdoviridae family of the genus Lyssavirus^1^. Transmission typically occurs via the saliva of a rabid animal, most often through bites, although cases of transmission through organ donation have been reported^1^. 

Rabies, along with 19 other conditions, is classified as a neglected tropical disease. The World Health Organization (WHO) has prioritized rabies for elimination, aiming to achieve zero human deaths from dog-mediated rabies by 2030^2^. In Brazil, prior to the 1980s, before the implementation of animal control and dog mass vaccination programs, human rabies (HR) cases were rampant^3^. During and after the COVID-19 pandemic, a sharp increase in HR cases and deaths has been reported worldwide^4^. Brazil has experienced a resurgence of HR in some states, such as Santa Catarina (after 38 years) and the capital, Brasília-DF (after 45 years)^5^. In this context, we aim to briefly discuss the main aspects of rabies prevention, surveillance, circulation, and behavior during the COVID-19 pandemic, both globally and in Brazil. 

## RABIES EPIDEMIOLOGY

According to the WHO, over 59,000 human deaths related to rabies are reported annually across more than 150 countries^5^. Most of these deaths occur in Asia and Africa^5^. HR remains endemic in developing countries within these continents. India accounts for 59.9% of rabies deaths in Asia, representing 35% of all rabies deaths globally. In 2020, India had the highest number of deaths (n=268), followed by Indonesia (n=40) and Sri Lanka (n=31)^6^. During the peak of the pandemic in 2021, the WHO data revealed that Indonesia (n=32) and Cote d’Ivoire (n=15) reported the most cases, as surveillance systems were compromised and redirected to COVID-19^6^. 

Globally, unvaccinated domestic dogs remain the primary cause of most HR cases. However, countries such as Brazil have observed shifts in animal transmission, with a predominance of wild animals, largely attributable to mass dog vaccination campaigns^7^ ^,^ ^8^. HR from dog bites was eliminated in the United States during the 1980s^8^. More recently, Mexico achieved canine rabies eradication in 2018^9^. Consequently, the epidemiology of rabies in these countries has shifted, with wild animals such as bats and skunks now being the main sources of HR^10^. In Brazil, common wild reservoirs include bats (Chiroptera) and wild monkeys, such as *Callithrix jacchus* ^11^ ^,^ ^12^. Bats are, in fact, the most frequent reservoirs of the rabies virus (RABV). In African countries, wild canids and domestic dogs continue to be significant RABV reservoirs^13^. 

## RABIES WORLDWIDE

## COVID-19 IMPACT ON POST-EXPOSURE PROPHYLAXIS WORLDWIDE

Deaths from rabies are preventable through prompt post-exposure prophylaxis (PEP). In Brazil for example, a significant reduction in rabies mortality rates over the past 40 years was observed following the implementation of canine vaccination campaigns and the intensification of PEP^14^ ^,^ ^15^. The United States and some European regions have historically reduced and eliminated dog-mediated HR, thanks to the availability of PEP with vaccination and immunoglobulin^16^. However, some discrepancies in PEP distribution and assessment remain^17^. The WHO Rabies Modelling Consortium developed epidemiological and economic models to investigate the effects of investments in PEP^16^ ^,^ ^17^. Predicting that more than 1 million deaths will occur in 67 rabies-endemic countries from 2020 to 2035, the study concluded that investing in post-exposure vaccines would be an extremely cost-effective intervention, capable of substantially reducing the disease burden and HR cases worldwide^18^.

Many countries in Africa and Asia continue to face challenges associated with limited access to PEP^19^. The COVID-19 pandemic significantly impacted essential health services for HR, raising concerns. Low- and middle-income countries, already struggling with inadequate medical supplies for HR prevention, saw their situations deteriorate^3^ ^,^ ^4^ ^,^ ^9^ ^,^ ^13^. The pandemic introduced additional strain on overburdened healthcare systems, with numerous medical centers either shutting down or reallocating resources to combat COVID-19. In Pakistan, rural regions faced an abrupt scarcity of rabies vaccine and immunoglobulin, leading to the death of a 10-year-old boy^19^. Patients in need of medical care and PEP found it difficult to locate urban centers offering vaccination^9^ ^,^ ^19^. This shortage at government facilities compelled patients from rural areas to turn to private providers for vaccination^19^. A similar situation unfolded in Bhutan, where a rabies death was reported in 2020-the first since 2016, due to COVID-19-related movement restrictions and the suspension of routine dog vaccination campaigns along Bhutan’s southern border^20^. While stringent paper verification processes hindered human cross-border movement, dogs crossed freely. 

The COVID-19 pandemic disrupted pre-established services for the prophylaxis and management of bite victims^3^ ^,^ ^4^ ^,^ ^10^. The sudden outbreak also hampered vaccine supply and prevented dog bite victims from seeking PEP due to travel restrictions, lockdowns, and the closure of anti-rabies clinics^10^. Additionally, fear of COVID-19 led some patients to avoid seeking care after animal exposures and accidents^5^. 

Conversely, HR-free countries such as France observed a dramatic decrease in rabies PEP demand in 2020 in the Ile-de-France region compared with the previous three years. Most cases in France are travel-related, given its status as a HR-free region. The reduced demand for HR vaccines is believed to be a direct consequence of movement restrictions^21^. This decline in PEP demand during the pandemic was a global phenomenon, particularly among travelers. Before 2019, PEP requests among international travelers had risen over 20 years from 0.7% (1998-2002) to 3.6% (2013-2018)^22^. The COVID-19 restrictions also led to a decrease in HR cases reported by travelers during 2020-2022. There was only one case of imported rabies, diagnosed in a Philippine migrant to Japan in 2020^23^. 

The pandemic exacerbated the issue of vaccine disinformation and misinformation. Sri Lanka, an Asian country endemic with HR, has seen a significant decrease in human rabies deaths over the last four decades, from a peak of 377 cases in the 1970s to 31 in 2020^6^ ^,^ ^24^. Despite this progress, the COVID-19 vaccine was not administered to individuals receiving the anti-rabies vaccine, and vice versa, due to health workers’ reluctance to enroll patients in vaccination programs in Sri Lanka. To our knowledge, there is no contraindication for administering the COVID-19 vaccine to patients undergoing PEP, or for providing PEP to those receiving the COVID-19 vaccine. In Brazil, the use of well-established vaccines in the National Vaccine Program declined due to hesitancy associated with the COVID-19 vaccine^25^. Data on PEP acceptance in Brazil are limited, and the potential impact of vaccination hesitancy on patients requiring PEP for HR cannot be discounted. 

## COUNTRIES WITH INCREASING INCIDENCE OF RABIES DURING THE PANDEMIC

Several countries experienced a resurgence of HR following the COVID-19 era. In Iran, the Department of Zoonotic Diseases at the Center for Communicable Diseases Control and Prevention reported 16 human rabies cases in 2021, compared to only six in 2018^4^. They hypothesized that the pandemic led to an increase in stray dog populations and the creation of dog vacuum areas, as well as disrupted rabies surveillance and a rabies vaccine shortage, thereby increasing rabies incidence in these areas^4^. Bhutan, another Asian country with a significant HR burden, faced a similar situation. To control the spread of COVID-19, Bhutan closed its borders. However, these measures did not prevent the escalation of rabies. Free-roaming dogs continued to cross borders, resulting in a resurgence of HR cases after years without a single reported incident^4^. 

In Latin America, significant progress has been made toward the elimination of canine rabies over recent decades. Mass dog vaccination has been a key strategy. However, COVID-19 has disrupted these efforts, compromising the prospects for elimination across the region. In Peru, Raynor et al. have indicated that reduced canine vaccination coverage, coupled with diminished surveillance, could precipitate a rapid increase in canine rabies cases within months. Surveillance data from late 2020 and early 2021 confirm an uptick in rabies cases in Arequipa, Peru^26^. Table 1 shows the countries with the highest number of HR cases during the COVID-19 pandemic by continent. Many countries experienced surveillance challenges for HR at the height of the COVID-19 pandemic, with a lack of data in 2021^6^. Furthermore, underreporting is a concern, as resources were diverted to address COVID-19^6^.

**TABLE 1:** Countries with the highest number of HR cases per continent notified during 2020-2021 according to WHO^6^ and SIRVERA^27^.

Country/Year	2020	2021
India (South Asia)	268	No data
Indonesia (Southeast Asia)	40	32
Sri Lanka (South Asia)	31	No data
Bangladesh (South Asia)	24	No data
Côte d-Ivoire (Eastern Africa)	18	15
Russia (North Asia)	7	0
Cuba (Central America)	3	1
Brazil (South America)	2	1
Mexico (North America)	1	0
Bolivia (South America)	1	5
Haiti (North America)	1	2
Colombia (South America)	1	1
United States (North America)	0	5

## RABIES IN BRAZIL

### ● During the COVID-19 Pandemic

Between 2010 and 2017, Brazil reported 188 HR cases, with a progressive and marked decline over those years^14^ ^,^ ^15^. From 2018 to 2021, only 15 HR cases were identified, most occurring before the COVID-19 pandemic. In 2020-2021, three cases were reported: one in Paraíba by AgV2 in 2020, one in Rio de Janeiro by AgV3 in 2020, and another in Maranhão by AgV2 in 2021^28^. As the pandemic abated, new HR cases emerged. During 2020-2021, most HR publications in Brazil focused solely on RABV identification in wildlife, with no cases reported^29^. In 2022, a rabies outbreak occurred in an indigenous village amid the pandemic’s decline^30^.

### ● After the COVID-19 Pandemic

**FIGURE 1:**
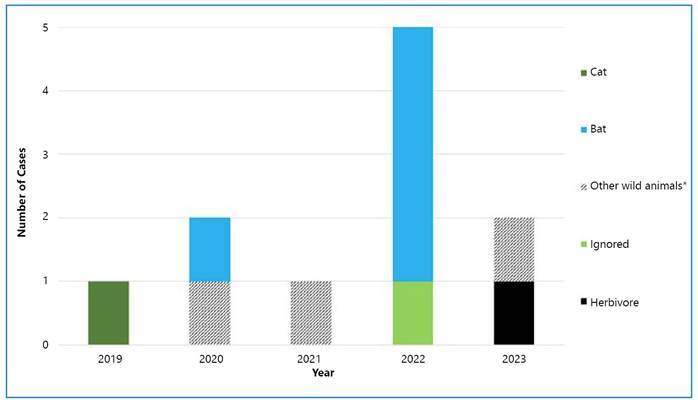
Cases of Human Rabies by Animal Species during the COVID-19 pandemic, 2019-2023, Brazil. *Wild canids, marmosets, and others.

After COVID-19 subsided in 2022, five cases were reported throughout the year; four were attributed to bats (Chiroptera), with the AgV3 variant identified in each instance. Conversely, in the first four months of 2023, two cases have already been reported: one in Minas Gerais and another in Ceará^31^ ^,^ ^32^. The former involved a 60-year-old man who sustained injuries from a bovine, in which the AgV3 variant was detected, while the latter concerned a 36-year-old man bitten two months prior by a non-human primate (*Callithrix jacchus*), potentially due to the new marmoset-related variant (AgV new). Ceará had not reported any cases of HR for seven years, with the most recent incident in 2016 involving a bat bite^14^ ^,^ ^15^. In 2022, an HR outbreak among indigenous children in Minas Gerais, Brazil, garnered the attention of health authorities. This outbreak resulted in the deaths of four indigenous children and marked the first recorded outbreak among this population, occurring post-pandemic^32^. Figure 1 depicts the distribution of HR cases by animal species during the COVID-19 pandemic in Brazil.

### ● Rabies surveillance systems in Brazil

Human rabies surveillance involves monitoring animal bites, reporting suspected and confirmed rabies cases in the community, documenting rabies deaths, and evaluating anti-rabies vaccine and serum coverage. Brazil employs the Information System for Notifiable Diseases (SINAN) to record patients seeking PEP after contact with animals suspected of harboring rabies^33^. Despite most efforts being redirected to the pandemic, surveillance systems like SINAN continued to operate, maintaining notifications and general surveillance^33^. However, the capacity to detect and diagnose HR likely diminished as the health system became overwhelmed, prioritizing the prevention of COVID-19-related human deaths^34^. Additionally, other surveillance methods, such as conventional autopsy may have been compromised, as most cases confirmed as COVID-19 were not subjected to autopsy to confirm or exclude other causes unrelated to *SARS-CoV-2, including* HR^34^. 

### ● Current situation of Rabies Prophylaxis in Brazil

Before the establishment of the National Rabies Prophylaxis Program (PNPR) in 1973, the majority of HR cases were attributed to dog bites. In that year, the Pan American Health Organization (PAHO) and the Ministry of Health initiated systematic rabies control measures. The PNPR’s strategy included mass dog vaccination, animal capture, laboratory diagnostics, epidemiological surveillance, health education, and preventive treatment for HR. Between 1980 and 1990, the implementation of mass dog vaccination and post-exposure prophylaxis (PEP), along with the organization of health services, led to a significant reduction in HR and canine rabies in Brazil^35^. Data from 2014 to 2019 showed 4,033,098 anti-rabies medical consultations, averaging about 672,183 annually. The typical profile of patients seeking evaluation for HR incidents were: male, under 19 years of age, residing in urban areas, and having been bitten by dogs^36^. A concerning observation is that the prophylactic procedure was adequately used in only an average of 57.8% of cases^36^.

### ● Social distance and Isolation

Social distancing and isolation were primary strategies during the COVID-19 pandemic in Brazil. The country’s first lockdown was decreed in March 2020^37^. However, adherence varied among the different states. The precise effect of social isolation on the growth or development of the stray dog population remains unclear. Nevertheless, it is believed that social isolation may have contributed to a reduction in human rabies cases. Notably, with social isolation, most animal-related incidents occurred at the household level involving domestic pets, obviating the need for rabies vaccination or immunoglobulin administration. 

## RABIES IN THE POST-COVID-19 ERA

Following the COVID-19 pandemic, several countries experienced a surge in rabies and HR outbreaks. Afghanistan, India, Lebanon, Sri Lanka, the Philippines, and South Africa reported rabies outbreaks shortly after the conclusion of the COVID-19 pandemic^20^. These increases in incidence have been linked to various factors, including the disruption of mass dog vaccination campaigns and decreased availability of HR services. 

In 2022, India reported a significant number of fatalities due to the disease (n=63). Additionally, there has been a worldwide surge in dog bite incidents, with reports suggesting increased aggression in stray dogs post-pandemic^20^. The Philippines reported an alarming number of HR cases compared to those reported in other countries (n=222). The causes of this rise remain unclear; however, the Philippines has recently encountered problems with counterfeit vaccines meant to protect patients^38^. Table 2 displays the countries with the most significant resurgence of HR cases globally.

**TABLE 2:** Rabies cases identified in some countries after COVID-19 pandemic subsided in 2022^27^ ^,^ ^39^

Country	Number of Cases (Nº)	Nº/ Per 100,000 inhabitants
Philippines	222	0,19
India	63	0,004
Afghanistan	10	0,02
Sri Lanka	12	0,05
Brazil	5	0,002
Mexico	4	0,003
Lebanon	2	0,03
South Africa	2	0,003
Yemen	2	0,006
Bolívia	1	0,008
Haiti	1	0,01

## DISCUSSION

Several factors have been implicated in the resurgence of human rabies (HR) after the COVID-19 era. During and after the COVID-19 pandemic, many neglected tropical diseases (NTDs), including rabies, suffered due to a global deficit in confidence and resources for essential prevention and epidemiological control. Specifically for rabies, the cancellation or disruption of dog vaccination campaigns, vaccine shortages, reduced animal testing, an increase in the stray dog population, and a rise in animal bites have all contributed to its resurgence. The interruption of dog mass vaccination campaigns between 2020 and 2021 may have played a role in the post-pandemic resurgence of HR^39^. Furthermore, the costs and concerns about COVID-19 transmission led to many dogs being abandoned and left without food, exacerbating the increase in strays and, consequently, the transmission of rabies^39^. Indeed, the pandemic underscored the need for ongoing rabies surveillance in dogs and the importance of maintaining continuous strategies that should not be interrupted worldwide.

The pandemic has led to numerous adverse financial consequences for the population, reducing access to medical care. Generally, during this period, certain healthcare sectors became overwhelmed and could not provide adequate care or report high-risk animal bites. In Brazil, the situation mirrored this, with the reemergence of rabies cases serving as a warning and underscoring the need for preparedness and control in addressing such incidents^32^. 

Data have shown the risks associated with neglecting one disease to focus on another. Historically, HR has posed a challenge to control and continues to be a disease with a 100 % fatality rate. In Brazil, the intensification of canine and feline rabies surveillance, along with the control measures implemented over the past 30 years, has led to a significant reduction in human rabies mortality rates. The remaining cases are predominantly sporadic and accidental^3^ ^,^ ^5^. The resurgence of HR cases should serve as a warning to resume control strategies with the same intensity and resources as those allocated before the pandemic.

According to the Ministry of Health, the COVID-19 pandemic in 2020 led to the suspension of dog vaccination campaigns in the states of São Paulo (SP) and Tocantins (TO), as well as in 219 other municipalities across various states. Despite the reduction in dog vaccination coverage during the pandemic, there was an increase in vaccination rates to 60.4% compared to 51.2% in 2019. However, this percentage was significantly lower than those reported in previous years, such as 82.2% in 2018^40^. To achieve the status of ‘rabies-free’ as outlined in ‘Zero by 30: The Global Strategic Plan to Prevent Human Deaths from Dog-Transmitted Rabies by 2030’^41^, Brazil and other countries must develop specific actions. Intensifying dog mass vaccination campaigns is likely one of the essential strategies needed to reach the goal of zero human rabies (HR) cases. With over fifty years of experience in rabies control programs, Brazil’s current main challenge is addressing wild rabies rather than canine rabies^3^.

In Brazil, seven variants have been identified: variants 1(AgV1) and 2 (AgV2), isolated from dogs; variant 3 (AgV3), from the vampire bat (*Desmodus rotundus*); and variants 4 (AgV4) and 6 (AgV6), from insectivorous bats (*Tadarida brasiliensis* and *Lasiurus cinereus*)^42^. Two additional variants, recently discovered in *Cerdocyon thous* (bush dogs) and *Callithrix jacchus* (white-tufted marmosets), had not been previously detected. Although AgV2 remains a commonly found variant in *Cerdocyon thous*, the most pressing concern regarding rabies in Brazil is the presence of the wild variant AgV3. The last reported cases in canines, identified between 2014 and 2016, were associated with variants 1 and 2 (AgV1 and AgV2), which may reflect improved control measures, including targeting stray dogs and widespread dog vaccination^43^. However, the impact of COVID-19 on mass dog vaccinations could potentially lead to a resurgence of canine rabies cases in the country. 

In summary, the predominant factors linked to the resurgence of HR during and after the COVID-19 era globally include: (1) Disparities in PEP access amid the COVID-19 pandemic; (2) Shift in priority from Rabies surveillance to COVID-19 response; (3) Decrease in mass dog vaccination campaigns; (4) Interruptions in the supply chain and availability of HR vaccines and immunoglobulins; (5) Unrestricted movement of free-roaming dogs; (6) Closure of healthcare facilities dedicated to HR management. 

## CONCLUSION

The COVID-19 pandemic redirected time, talent, experts, hospital centers, the health system, and resources away from NTDs. Access to PEP, surveillance, and control of roaming dogs have declined worldwide, particularly in low-income countries endemic for HR. Following COVID-19, a resurgence of HR has been observed globally, including in countries from Asia, Africa, and South America. Canine variants appear to be the primary issue in Africa and Asia, while wild variants are more significant in South American countries. There is a clear need for action and reinforcement of HR control measures to prevent future outbreaks. Despite being a notifiable disease, rabies in both animal and human populations remains underreported. Rabies will continue to pose a threat to humans as long as the infection persists in animals, particularly dogs, worldwide. Mass dog vaccination strategies are essential, especially in African countries, to prevent HR. Investments at national and global levels must be resumed for the prevention, surveillance, and control of human and canine rabies^3^. Brazil is not immune to the need for reinforced control strategies to contain human and canine rabies, with the main challenge relating to wildlife and their rabies variants. HR cases continue to be identified in the country, presenting a life-threatening risk to its citizens. Disseminating knowledge about HR within society is crucial to prevent contact with rabies-transmitting wild animals. The solution to HR involves various areas of expertise, incorporating the One Health approach, social education, and public health measures.
